# Recruitment and Attrition for Panel Surveys of Hard-to-reach Populations: Some Lessons from a Longitudinal Study on Undocumented Migrants

**DOI:** 10.1177/1525822X231210415

**Published:** 2023-11-09

**Authors:** Aline Duvoisin, Jan-Erik Refle, Claudine Burton-Jeangros, Liala Consoli, Julien Fakhoury, Yves Jackson

**Affiliations:** 1 Swiss National Center of Competence in Research (NCCR) “LIVES – Overcoming Vulnerability: Life Course Perspectives”, 27230 University of Geneva, Geneva, Switzerland; 2 Center for the Interdisciplinary Study of Gerontology and Vulnerability, 27230 University of Geneva, Geneva, Switzerland; 3 Institute of Sociological Research, 27212 University of Geneva, Geneva, Switzerland; 4 27230 Geneva University Hospital and University of Geneva, Switzerland

## Abstract

Conducting research among hard-to-reach populations is a difficult endeavor because some of their characteristics are known to be associated with survey nonresponse and panel attrition. In the case of the Parchemins study, which followed undocumented migrants over their process of regularization and during the first years of regularized life in Geneva, we underscore the difficulties in recruiting and keeping respondents who come from such a hard-to-reach population. Factors hindering their participation include the fear of being denounced as undocumented, missing time due to high workload, health issues, or language problems. Using unique data from the recruitment and the follow-up processes, we demonstrate that investing high resources and time is particularly beneficial to reach such a population and to reduce attrition over successive data collection waves. In addition, we present the strategies adopted to draw a convenient sample from our targeted population, which mainly relies on generating trust.

## Introduction

Research on hard-to-reach populations (Shaghaghi et al. 2011) or vulnerable populations (van Liempt and Bilger 2012) is difficult as the targeted people often live in precarious conditions, are exposed to different forms of discrimination and express fears usually not present in the general population. For undocumented migrants, general accessibility and fear of denunciation are just a few of the problems to deal with. Our article examines how these problems were addressed in the Parchemins study, a longitudinal project that followed undocumented migrants in their process of regularization and during the first years of regularized life in the Swiss canton of Geneva. The regularization was part of the exceptional “Papyrus” program implemented between 2017 and 2018. This pilot program was exceptional in the sense that it was the first of its kind in Switzerland, implemented in a single Swiss canton and took place in a period of restrictive immigration policies in Western Europe. It resulted from a decade-long elaboration process involving multiple stakeholders in Geneva and the federal administration that led to the definition of a set of operational regularization criteria without modifying the existent legal framework. Finally, it gave a prominent role to community organizations acting as gatekeepers into the administrative process. We followed migrants who applied for a residence permit, as well as some others not eligible for regularization and remaining undocumented, through a four-waves panel study to assess the consequences of the legal status change on living conditions and health status.

We address two research questions: First, how to recruit a panel among a hard-to-reach population? Second, how to maintain such a panel? Both questions are highly related to how the target population is approached. The issues we address not only concern small panel surveys, but equally panel surveys of larger populations. Existing studies show that representation in general population panel research is associated with social integration and less integrated groups tend to be systematically under-represented (Lipps 2007).

In this article, we argue that more time and larger efforts invested are important to better reach such populations. We also assess how a few predictors relate to participation using a discrete regression model. We show that the participation rate does not just decline with time, since this relation is not linear.

First, the article defines what characterizes hard-to-reach populations and provides a synthesis of what other studies found effective to avoid attrition. In the second part, we outline how our longitudinal study with undocumented migrants unfolded and what can be learned from the strategies we used.

## Recruitment and Attrition of Hard‐to‐reach Populations

### How Are Hard‐to‐reach Populations and Vulnerable Populations Defined?

Members of hard-to-reach populations are difficult to recruit for a number of reasons: They are either part of a small population, hard to identify, have characteristics associated with social disapproval, for which no sampling frame exists, whose members do not wish to be associated with the target population, or for which the absence of information on their situation implies inadequate sampling strategies (Marpsat and Razafindratsime 2010). Others define a hard to reach population as groups with “low SES [socio-economic status], members of certain ethnic minorities, and persons with low levels of literacy” (Freimuth and Mettger 1990:232). A low SES can relate to precarious living conditions (Rothenbühler and Voorpostel 2016), but also to low educational levels. Stoop (2005) adds that SES interacts with people's willingness to participate, mentioning that migrants belong to a group with lower response rates. Those characteristics are similar to those of other hard-to-reach populations like prisoners (Fahmy et al. 202).

Literature on hard-to-reach populations is often linked to research on vulnerable populations, such as migrants. The term ‘hard-to-reach’ relates to methodological questions, while vulnerable populations are defined on a more theory-driven basis as described below. However, over-researched people, or people living in remote areas, can also be hard-to-reach (Shaghaghi et al. 2011), suggesting that hard-to-reach populations are not automatically vulnerable.

Vulnerable populations describe individuals who are marginalized or faced with poor living conditions and discrimination. Migrants are described as a vulnerable group, particularly in the case of irregular migration and precarious situations (van Liempt and Bilger 2012). Difficult and often hazardous work conditions in the informal sector affect the living conditions of undocumented migrants (Woodward et al. 2014). Besides, their habits are not well known, due to camouflage and adaptation strategies that make them barely visible. Their insufficiently known characteristics, heterogeneity, and absence from official census records thus make them particularly difficult to study (Chauvin and Garces-Mascareñas 2014:424). The frequent lack of any estimate of their number is an additional challenge (Wesson et al. 2017).

### How to Recruit and Maintain Hard-to-reach Populations

Enticott et al. (2017) implemented an extensive literature review on studies that claimed to be representative of asylum seekers and refugees, a group showing some similarities with undocumented migrants such as language barriers and low SES. They said that “an a priori aim to recruit a representative sample; a reliable sampling frame; recording of response rates; implementation of long recruitment periods; using multiple non-probability sampling methods; and, if possible, including a probability sampling component” was crucial to achieve a representative sample (Enticott et al. 2017:14). Scholars also highlight the relevance of community engagement, as community organizations often have already gained the trust of potential participants (Ellard-Gray et al. 2015; Enticott et al. 2017). However, a community-based sampling may create a bias, reaching only specific sub-populations who attend those locations (Ellard-Gray et al. 2015; Meyer and Wilson 2009).

Snowball sampling is often used with hard-to-reach populations. This technique uses interpersonal trust that unfolds once the first contact is achieved (Atkinson and Flint 2001; Handcock and Gile 2011; Sadler et al. 2010). However, only connected individuals participate (Sadler et al. 2010). Over time, alternative approaches like respondent based sampling and spatial sampling emerged (Handcock and Gile 2011). Others propose a combination of venue-based and time-based sampling where participants are recruited at locations known to be frequented by hard-to-reach populations and this over different days and times (Muhib et al. 2001).

A number of barriers in reaching hard-to-reach populations have been described. Ethnic groups are particularly concerned about the potential uses of the collected data, associated with social stigma and fear of denunciation (Ellard-Gray et al. 2015; Mühlböck et al. 2018; Rothenbühler and Voorpostel 2016). Language is another obstacle, and evidence show that translated questionnaires improve response rates (Stoop 2005). In the absence of a residence permit, undocumented migrants tend to use camouflage techniques to avoid being identifiable by authorities (Chauvin and Garcés-Mascareñas 2014), a problem that can also affect researchers when, for example, participants’ mobile numbers change. Similar issues were identified in research with other hard-to-reach populations (Fahmy et al. 2022). Difficulties in contacting such a population are increased by its members’ working profile, usually with a high workload across multiple workplaces. Nevertheless, topics that are important to interviewees contribute to increased response rates (Stoop 2005).

Panel studies present a double challenge. Beyond recruitment, it is important to maintain people on the panel. Thus, it is not surprising that panel studies with hard-to-reach populations are extremely rare. One of the few available examples is an Australian study on humanitarian migrants that used different sites for initial recruitment (De Maio et al. 2014). Rothenbühler and Voorpostel (2016) and Lipps (2007) address attrition of panels for vulnerable groups, analyzing the Swiss Household Panel dropout rates. An Austrian longitudinal study on young unemployed underlines the efforts needed to keep participants in the panel (Mühlböck et al. 2018).

Various causes of panel attrition for such populations have been documented. Respondents facing more changes in life are more likely to drop out in panel studies (Lipps 2007; Voorpostel and Lipps 2011). Furthermore, mobile people—such as migrants moving between regions or countries (Consoli et al. 2022)—have higher dropout rates in panels (Watson 2003). Low-income levels or short-term residence permits are also associated with higher attrition (Rothenbühler and Voorpostel 2016). Finally, if the first-wave data collection was stressful, participants are more likely to drop out (Mühlböck et al. 2018).

Literature on panel recruitment and attrition for vulnerable and hard-to-reach populations suggests that it is particularly difficult to achieve panel data because of low socioeconomic status, time constraints, but also availability of possible recruitment places and trust of participants. In the following, we address how the Parchemins study overcame those barriers.

## The Parchemins Study: A Panel Survey among Undocumented Migrants in Geneva

The study started in 2017 in Geneva, when local authorities decided to implement a two-year regularization program (“Operation Papyrus”). This public policy provided a renewable one-to-two-year residence permit to undocumented workers meeting specific requirements: a stay of 10 years (five years for families with children at school), an A2-French proficiency, financial autonomy, absence of criminal prosecution, not being national of a country of the European Union or the European Free-Trade Area, and never having been an asylum seeker.

The Parchemins study aimed to measure regularization impacts on living conditions, health, and well-being of migrants gaining a residence permit (Jackson et al. 2019). It followed a group of undocumented migrants undergoing regularization and a group of migrants who could not apply and thus remained undocumented. Both groups were recruited to ensure at least partial comparability; an additional requirement for participation in the study was a residence duration of more than three years (Jackson et al. 2019). The panel survey consisted of four successive waves conducted between 2017 and 2022, at an approximately one-year interval. The baseline data were collected between November 2017 and October 2018, when migrants applied for regularization. Prior to our study, it had been estimated that about 10,000–15,000 undocumented migrants live in Geneva, representing 2%–3% of the overall population of the canton (Morlok et al. 2015). This population consists mainly of women from Latin America and the Philippines working in the domestic sector (Jackson et al. 2019).

### Initial Recruitment Strategy

The initial sample was mainly recruited through non-governmental organizations that acted as gatekeepers for the residence permit application. Associations filtered applicants on eligibility criteria before helping them submit an application. Some participants were recruited through the Geneva University Hospital, providing primary care to undocumented migrants. To inform potential participants, posters about the study were hung at the associations, at the dedicated medical unit and at additional contact points, as well as on social media (Facebook). These posters mentioned both the University of Geneva and the Geneva Hospital as co-sponsors of the study, to garner additional trust. As recruitment was based on confidence building, this accompanying measure was deemed useful to not only gain the trust of gatekeepers, but also of members of the target population.

The study largely benefited from the support of these community partners who acted as entry points (mainly organizations that work on the defense of migrant rights and unions). They have been involved as partners of the study—being also partners of the government procedure. This allowed a twofold recruitment strategy. First, interviewers recruited potential participants on site during opening hours of the medical unit and the gatekeepers’ associations. Second, these partners directly informed potential participants and, if they agreed to particpate, provided us with their contact information.

**Figure 1.**
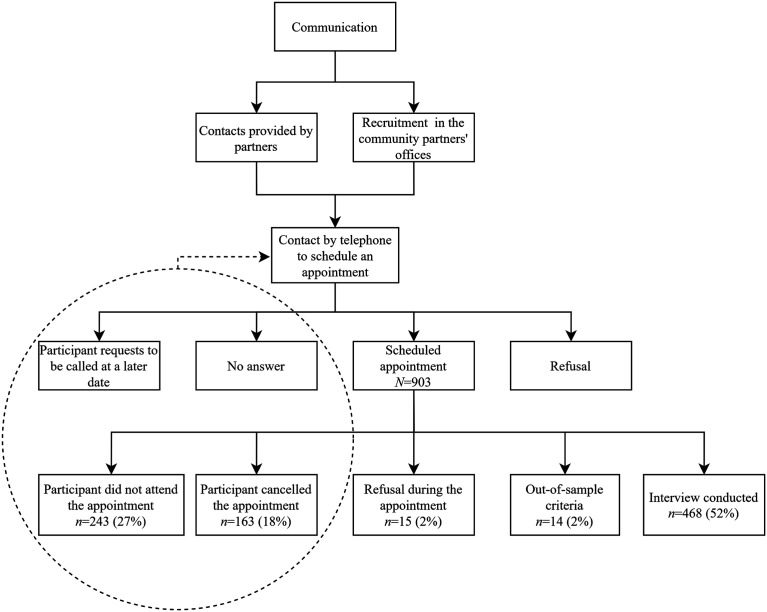
Recruitment process and outcome of the scheduled interviews during recruitment process.

For the initial recruitment process, interviewers spent approximatively 500 hours in the community partners’ offices obtaining contacts and 400 hours on the telephone to schedule interviews with potential participants. As shown in Figure 1, we managed to schedule 903 interviews, of which 18% were canceled and 27% did not attend. The first data collection wave reached 468 participants, equaling 52% of appointments that had been scheduled.

We used computer-assisted personal interviews (CAPI) throughout study waves. Face-to-face interviews were important because they increase chances that respondents finish the survey (Mühlböck et al. 2018). Waves 3 and 4 were conducted after the outbreak of the Covid-19 pandemic, so part of the interviews were conducted via video conferencing.

Questionnaires and interviewers were multi-lingual to ensure that participants could express themselves in a language they felt comfortable with. Interviews were conducted in the main languages spoken by this population: French, English, Spanish, and Portuguese. Participants could also choose the location and the time (including after regular working hours or during the weekend) of the interview to create flexibility and to ensure that participation was manageable and agreeable for them. Participants were informed that their participation would not increase chances of regularization. After each encounter, participants efforts were acknowledged by offering a symbolic non-monetary gift (USB-key, cloth bag, etc.), and the research team maintained friendly relationships by sending seasonal greetings at the end of each year.

The study was approved by the local ethics board and at the first interview, participants gave their informed consent for participation, while being informed about the longitudinal character of the study, hence that they would be contacted again later.

Against the pre-existing estimate of 10,000–15,000 undocumented migrants living in Geneva (Morlok et al. 2015), we are unable to determine whether our sample is representative of the entire population of undocumented and newly regularized migrants. Nevertheless, we strived to diversify the profiles of participants to represent the various countries of origin. As the characteristics of the undocumented migrant population are unknown, we are unable to construct weights allowing us to generalize our findings to Geneva-based undocumented migrants.

Some ex-post validation of our data can be done against the published official numbers provided by the regularization program. Overall, 2,390 persons were regularized at the beginning of 2020, including many families (Ferro-Luzzi et al. 2019; République et Canton de Genève 2020). More than 700 applications were still under evaluation at that time. Given that we surveyed 468 migrants in total, including 316 already documented at the beginning and 52 who got regularized over the course of the study, we estimate that our data cover a relatively high share of the at least 1,651 adult individuals (as we do not include children) who obtained a residence permit through the Papyrus Operation (Ferro-Luzzi et al. 2019). While we cannot guarantee that our data represent undocumented migrants in Geneva, it is very likely that its composition comes close to the population of newly documented migrants in the framework of the Papyrus operation.

### Attrition and Longitudinal Follow-up Strategy

At the end of each interview, participants were asked if they accepted to be contacted again for the following wave. If they agreed, they were asked to confirm their contact details (phone number and/or email). This was important as this highly flexible population often changes phone numbers and email addresses. In addition, not having a residence permit makes it impossible to have a fixed phone number subscription.

During the successive waves of data collection, interviewers used multiple techniques to reach those who had given their consent to participation in a later wave. They contacted the participants primarily by phone, but also by email or text messages using applications such as WhatsApp, Viber, or Telegram, when they did not receive an answer after several phone attempts. This multi-contact strategy enhanced the recruitment and re-contacting chances as some participants turned out more likely to respond to WhatsApp messages than to a phone call. Interviewers also varied the timing of their calls. They were instructed extensively beforehand and were asked to record all their contact efforts with participants. This led to the database used for the analyses presented below.

**Figure 2.**
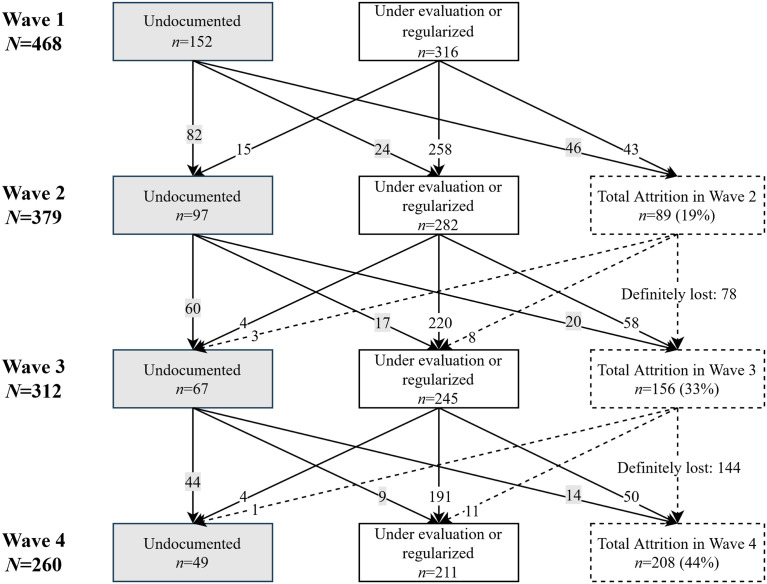
Number of participants by wave and attrition.

As a result of attrition, the panel size was reduced by 44.4% between wave 1 and wave 4. This corresponds to a total attrition rate of 19% in wave 2 towards 33% in wave 3 and 44.4% in wave 4. Compared to the Swiss Household Panel (SHP), a general population panel, attrition in our study is lower than in the refreshment sample of SHP (28%–29% for each early wave, Voorpostel et al. 2021). Figure 2 shows the decline in participation across all waves.

### Evaluating the Efforts to Reduce Attrition

Due to our tracking system, we have a unique dataset on the number and the timing of contacts. Because of the Covid-19 pandemic, the following analyses only include wave 2, since waves 3 and 4 do not represent normal contacting situations. Indeed, the living conditions of respondents changed substantially in more recent waves and the number of contacts needed to organize an interview can hardly be compared with the pre-Covid environment.

At the end of the first wave questionnaire, 459 (98.1%) participants agreed to be recontacted in the second wave. For the second wave, conducted between March 2019 and February 2020, interviewers' persistence and adaptability played a key role. However, further challenges emerged in wave 2. Establishing contacts was difficult, as some participants changed their phone number since the first interview. Some asked to be called back several days or weeks later as they were too busy at that moment or wanted to think about their willingness to participate. Even in cases of scheduled appointments, 11% did not show up and 26% canceled or reported interviews. Still, 63% of all scheduled interviews were successful. Explicit refusals for participation in wave 2 were rare as only 20 individuals (4.4%) expressed an objection to further participate in the study (in addition to the 1.9% who had objected to continue their participation at the end of wave 1).

Panelists in wave 2 were on average contacted 6.4 times. Compared to other surveys where about four calls achieved a near full participation rate (Mühlböck et al. 2018), our hard-to-reach population is far from those values. 34.8% of the participants in the second wave actually did the interview after five or more contact attempts.

Also, the time between each attempt was an important factor. We estimated the probability that the interview has not yet occurred for each day since the first contact attempt through a survival function. The estimated median survival time of 35 days means that for half of the panelists, up to 35 days elapsed between the first contact attempt and the interview (or an explicit refusal). Nineteen percent could not be reached at all, and the remaining 31% took up to five months after the first call to get an interview (or refusal).

As reported in other studies (Mühlböck et al. 2018), repeated contact attempts were effective and resulted in additional respondents even long after the first contact attempt. However, the costs in time and effort to achieve these additional responses were particularly high. Studies typically evaluate these costs against the relative benefits of additional efforts (Stoop 2005). We argue that these tradeoffs are different in general and hard-to-reach populations. In population studies such as household panels, underrepresented groups can be partially equalized by weighting, while the relative costs for recruiting additional respondents from underrepresented groups are rather high. In that case, the chance of realizing an interview decreases over time in a linear relation between time and participation. However, we observed that for hard-to-reach populations, this relation is not linear. This observation was possible because we purposively extended contact attempts over a long period of time, due to the high value of each additional interview.

We examined the relationship between the time (i.e., number of days) since the first contact attempt and the conditional probability for each respondent to participate in the second wave interview (i.e., the discrete-time hazard) (Supplementary). The estimated slope indicates that participation stopped decreasing after 60 days and remained stable before trending upward after several months. In other words, the probability that a person participates diminishes with ongoing time, but then increases slightly again after a longer time frame. To elaborate this finding, we tested whether other variables have an influence on continued participation in the study.

### The Non-linear Relation between Participation and Time

We use data collected in wave 1 and from fieldwork monitoring in wave 2, including a detailed assessment of the information collected by interviewers during the 2nd wave. The 459 participants who accepted to be re-contacted for wave 2 were considered as potential wave 2 participants from the day that the interviewer tried to contact them for the first time. Then observations were collected until the second interview was actually completed or the participant refused to continue participation. For the remaining cases, contacts were stopped at a minimum of 188 days after the first contact attempt. This maximum period of observation was defined by the research team, balancing the benefits and costs of additional attempts.

We conducted a discrete-time regression model with participation in wave 2 as the dependent variable (continuous) and including a mix of independent variables from waves 1 and 2. Time (days since first contact attempt) as well as time-squared are included as we are interested whether the non-linear relation between time-passed and participation is observed in a regression model. The analysis also includes the number of contact attempts during this second fieldwork as a measure of interviewers’ perseverance. Considering the association between attrition and past experiences in the study (Lugtig 2014), we include the duration of the interview in wave 1 (with normal interviews at 45–60 minutes, shorter at less than 45 minutes, and longer with more than 60 minutes) and the language of the first interview (French vs, Spanish, English or Portuguese). We also assess several socio-demographic variables derived from wave 1, including sex, age (44 and older vs. younger persons, based on the median age of the wave 1 sample) and education (tertiary education vs. primary and secondary levels). Living condition are controlled by including whether a demand for regularization was submitted in wave 1, employment, self-rated health (very good and excellent health vs. poorer health), participation in a club or association and an index of global satisfaction (continuous) including satisfaction with financial situation, satisfaction with accommodation, satisfaction with personal relationships, and satisfaction with life in general.

This discrete-time survival analysis aims at examining to what extent time elapsed since the first contact attempt, socio-demographic characteristics, living conditions and “past experience in the study” variables had an independent effect on participation in wave 2. Compared to other statistical methods, survival analysis can include both time-fixed and time-varying factors in the same model and can deal with right censoring data. Among the existing survival analyses, we conducted discrete rather than continuous-time analyses, as discrete-time analysis has no problem with ties (i.e., multiple events occurring at the same time point). Thus, it is compatible with the generalized linear model (GLM) framework. We fitted two models: The first only including the time and time squared to verify our hypothesis and the second including the additional factors. The complete overview can be found in the supplementary material and presents adjusted odds ratio (aOR) with confidence intervals (95% CI).

The regression analysis (supplement) shows that not only time, but also time-squared are significant explanatory variables for whether the contact was finally successful. This means that we observe high participation at the beginning, but equally more toward the end of the recruitment phase. This non-linear relation suggests that a longer time frame has a positive effect on participation. Nevertheless, we observed no effect of language on participation, which means that the proposed languages seem sufficient to mitigate attrition. The length of interviews was significant, showing that when the first interview was either very long or rather short, drop out for the following wave was more likely. Participants who applied for regularization in wave 1 have a higher chance of continued participation.

## Discussion

Our article aimed to outline strategies to reach and maintain hard-to-reach populations in longitudinal studies. Experience gained in the Parchemins study confirms the importance of confidence building, diversified recruitment locations and strategies, as well as approaching participants in different languages at initial recruitment. The same factors proved equally important for maintaining participation. In addition, our study confirms that many contacts augment participation rates among hard-to-reach populations as shown by others (Mühlböck et al. 2018). Using multiple contact channels like phone calls and messaging apps supports participation and reduces attrition. One of the lessons learned includes the importance of a high flexibility with regard to moments, forms of communication, and language of participation as well as planning a period of pre-interview contacts long enough to give participants the best opportunity to respond positively to the participation invitation at their convenience.

The increased participation after numerous contact attempts reveals the non-linear relationship between participation and time. Our study shows that a high-level of contacts can increase response rates substantially. It contrasts with current assumptions in the literature (Stoop 2005), according to which there is a linear relation between the time invested in contacts and the number of gained responses with the initial high rate of responses declining regularly over time. We argue that this does not apply to hard-to-reach populations to a similar degree. When extending the time frame and the number of possible re-contacts, we observe a curvilinear relation, with a rather rapid decrease followed by a reversing trend on the long term. In our study, we would have only kept about 65% of the population if we had stopped recontacting after the recommended number of four calls. Extending the number of contacts over time through multiple channels proves favorable. This finding supports earlier results on hard-to-reach populations where the number of efforts was deemed crucial for higher participation rates (Mühlböck et al. 2018). At the same time, fieldwork cannot be unconditionally extended for two reasons. First, important resources are often not available in the long run; second, multiplying contact attempts raises ethical concerns about the appropriate level of researchers’ perseverance against the necessary respect of participants’ unwillingness, reluctance, or inability to participate to additional data collection. Among the lessons learned is the need for a continuous operational and ethical evaluation of costs and benefits of efforts deployed toward additional participation and of conditions justifying to go beyond usual numbers of contact attempts.

General population surveys face less attrition problems as they reach representative samples earlier. However, our findings suggest that currently underrepresented sub-populations like migrants could be re-integrated by proposing longer and more flexible timings to them. Cost–benefit analyses would probably lead larger surveys to opt for weighting rather than investing more time in improving participation. We argue that studies focusing exclusively on hard-to-reach populations can reduce attrition by investing more resources and time in recruitment and follow-up. Yet this requires careful financial and operational anticipation at the time of the study plan development and fund raising. We recommend that researchers elaborate on the extra efforts needed to recruit and maintain hard-to-reach participants in a cohort study in their communication with funding agencies. Indeed, such a strategy represents significant costs in terms of human resources. In the case of Parchemins study, we had to gain additional resources to those obtained in the original research grant proposal.

Besides ascertaining the constant supervision of the recruitment efforts by a postdoctoral fellow, our heavy investment in recruitment has been supported by several cohorts of multilingual students. Their commitment provided flexibility regarding the timing and intensity of fieldwork that proved crucial to maintain our panel's participation as high as possible. Future research on hard-to-reach populations must consider the higher resource needs and consequentially higher costs.

The study achieved a rather good participation rate over time without proposing additional material benefits for participation. Incentivization increases response rates and decreases attrition. For a hard-to-reach population with limited economic resources, it may be particularly attractive. Incentives prove more useful to keep people on a panel than for initial recruitment (Stoop 2005). In the Parchemins study, we did not offer any financial incentive up front for the quantitative panel, however we acknowledged participants contribution after each interview with a small symbolic non-monetary gift. Incentives can substantially increase research costs and raise ethical concerns about the autonomy of participation. In our view, initial trust is what really matters, for the initial recruitment and the follow-up in a longitudinal design. Monetary incentives might have increased participation, but it would have created a different perception of the study. Additional studies should shed light on the possible trade-offs between trust-building and monetary incentives for hard-to-reach populations. This provides an additional lesson-learned from our study (i.e., the possibility to conduct such a study without giving material incentives by multiplying trust-building strategies). Such strategies should focus on direct trust building with the participants, as well as on indirect trust building via trusted actors, such as the community organizations in our case.

Our findings have some shortcomings. Notwithstanding several efforts, attrition remains relatively high, even if comparable to other panel studies. The decline in participation is higher from wave 3 onward, at a time when we had to cope with severe consequences of the pandemic for the participants (Burton-Jeangros et al. 2020). Some participants may have faced several life-changing events in the follow-up of the regularization so their participation in a panel study may not have been their first priority. Nevertheless, we believe that we generated a unique longitudinal database to document the living and health conditions of vulnerable migrants.

## Conclusion

Hard-to-reach populations are known to be linked to lower response rates and attrition in panel studies. While our study is no exception, we argue that such populations can be reached through intense efforts to build trustful relationships and the allocation of high resources in time and researchers for contacting potential participants. This similarly applies to maintaining those populations in the panel. Since the relationship between time for response and participation tend not to be linear for hard-to-reach populations, research teams need to carefully decide on duration of data collection and the resources that they are willing and able to invest. This is an important argument that funders should be attentive to.

## Supplemental Material

Supplemental Material - Recruitment and Attrition for Panel Surveys of Hard-to-Reach Populations: Some Lessons From a Longitudinal Study on Undocumented Migrants Supplemental Material for Recruitment and Attrition for Panel Surveys of Hard-to-Reach Populations: Some Lessons From a Longitudinal Study on Undocumented Migrants by Aline Duvoisin, Jan-Erik Refle, Claudine Burton-Jeangros, Liala Consoli, Julien Fakhoury and Yves Jackson in Field Methods
