# Supplementary material for: Recruitment and Attrition for Panel Surveys of Hard-to-reach Populations: Some Lessons from a Longitudinal Study on Undocumented Migrants
Source: Field methods. 2023 Nov 9;36(4):294–310. doi: 10.1177/1525822X231210415 (PMC11439582; doi:10.1177/1525822X231210415)
Supplement: Supplemental Material - Recruitment and Attrition for Panel Surveys of Hard-to-Reach Populations: Some Lessons From a Longitudinal Study on Undocumented Migrants [file sj-pdf-1-fmx-10.1177_1525822X231210415.pdf]

Annex

Figure 2: Scatterplot between discrete-time hazard and time since the first contact attempt

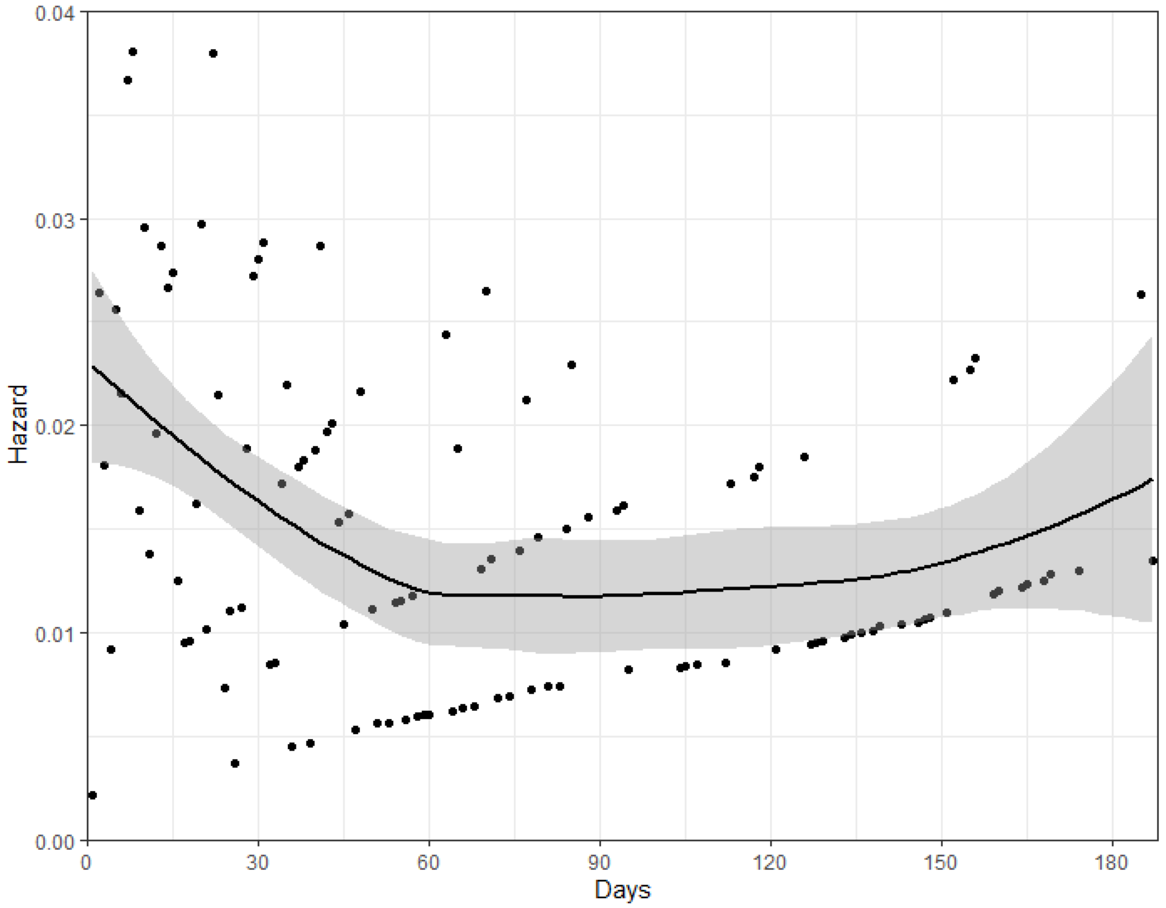

Table 2: Adjusted Odds-ratios for Discrete-time Regression Models on participation in Wave 2

	Exp (Est.)	95% CI	Exp (Est.)	95% CI
(Intercept)	0.03***	(0.02, 0.03)	0.01***	(0.01, 0.02)
Time	0.98***	(0.97,0.99)	0.98***	(0.97, 0.99)
Time²	1.0*	(1.00,1.00)	1.00*	(1.00,1.00)
Number of contacts attempts			1.06**	(1.01,1.12)
Duration first interview, short (ref= "normal")			0.74**	(0.57,0.95)
Duration first interview, long (ref= "normal")			0.76**	(0.58,0.99)
Interview in French (vs ESP/ANG/POR)			1.05	(0.83,1.32)
Sex (women)			0.93	(0.73,1.20)
Aged (44-73)			1.01	(0.80,1.27)
High education level (vs. Medium & low)			1.00	(0.78,1.29)
Application submitted in Wave 1			1.45***	(1.12,1.87)
Working in Wave 1			1.41	(0.91,2.20)
Bad health evaluation in Wave 1			1.25*	(0.99,1.56)
Participation in club or association			1.15	(0.90,1.47)
Satisfaction index			1.01	(0.99,1.02)

****p<0.01 **p<0.05 *p<0.1*

Note: Short interviews were under 45Min. , normal ones between 45 and 60 minutes and long interviews above 60 Min. ; n: 459, no. Of observations: 29767 for both models, r2 for the first model 0.03, for the second 0.04
